# Supplementary material for: Radiosensitising Cancer Using Phosphatidylinositol-3-Kinase (PI3K), Protein Kinase B (AKT) or Mammalian Target of Rapamycin (mTOR) Inhibitors
Source: Cancers (Basel). 2020 May 18;12(5):1278. doi: 10.3390/cancers12051278 (PMC7281073; doi:10.3390/cancers12051278)
Supplement: Supplementary file 1 [file cancers-12-01278-s001.pdf]

Supplemental Materials

Radiosensitising Cancer Using Phosphatidylinositol-3-Kinase (PI3K), Protein Kinase B (AKT) or Mammalian Target of Rapamycin (mTOR) Inhibitors

Kasun Wanigasooriya, Robert Tyler, Joao D. Barros-Silva, Yashashwi Sinha, Tariq Ismail and Andrew D. Beggs

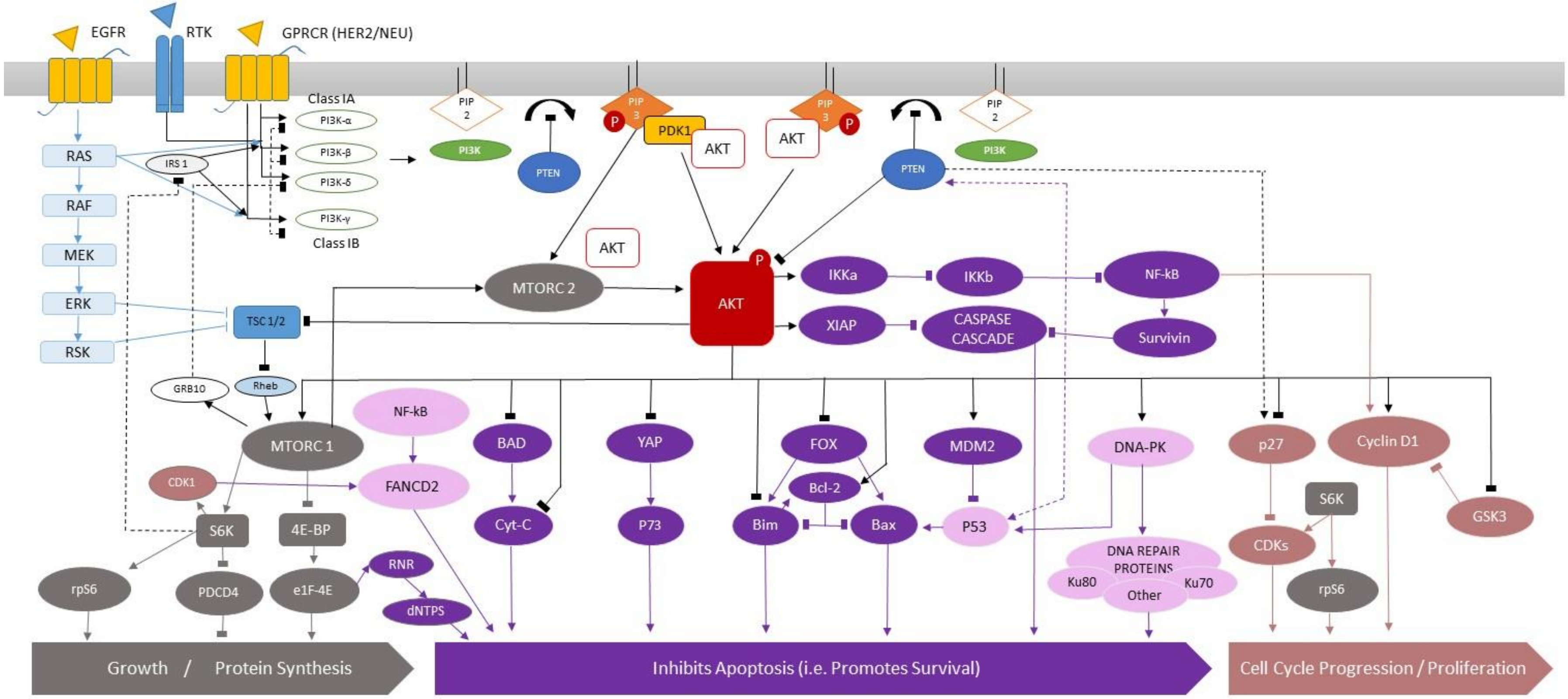

Figure S1. The PI3K/AKT/mTOR pathway activation leads to cell growth, increased protein synthesis, inhibited apoptosis, cell cycle progression and proliferation. **<u>Regulatory proteins:</u>** Phosphatidylinositol-3-kinase (PI3K) - class IA (α, β, δ) or class IB (γ), Phosphatidylinositol-4,5-bisphosphate (PIP2), Phosphatidylinositol-3,4,5-bisphosphate (PIP3), 3-phosphoinositide-dependent protein (PDK1), Tuberous sclerosis proteins 1 and 2 (TSC-1/2), RAS homolog enriched in brain (Rheb),

Growth factor receptor bound protein 10 (GRB10), Insulin receptor substrate 1 (IRS 1), Phosphatase and tensin homolog (PTEN), Protein kinase B (AKT), Receptor tyrosine kinase (RTK), G-protein coupled receptor (GPRCR), Epidermal growth factor receptor (EGFR). **Proteins involved in cell growth and protein synthesis:** Mammalian Target of Rapamycin (mTOR), S6 kinase beta-1 (S6K1), Eukaryotic translation initiation factor 4E (eIF4E)-binding protein 1 (4E-BP1), Programmed cell death protein 4 (PDCD4), Ribosomal protein S6 (rpS6). **Proteins involved in promoting cell survival and inhibiting apoptosis:** The IκB kinase alpha (IKKα), IκB kinase beta (IKKβ), nuclear factor kappa-light-chain-enhancer of activated B cells (NF-kB), X-linked inhibitor of apoptosis protein (XIAP), BCL2 associated agonist of cell death (BAD), Cytochrome C (Cyt-C), yes-associated protein 1 (YAP), p73, Forkhead box proteins (FOX), B-cell lymphoma 2 protein (BCL-2), BCL-2-like protein 11 (Bim), BCL-2-associated X protein (BAX), Mouse double minute 2 homolog (MDM2), DNA-dependent protein kinase (DNA-PK), Ku-80, Ku-70 Ribonucleotide reductase (RNR), Deoxynucleoside triphosphate (dNTP), Survivin, Caspase cascade proteins. **Proteins involved in cell cycle progression and proliferation:** Cyclin dependent kinase 1 (Cyclin D1), Glycogen synthase kinase 3 (GSK3), p27, Cyclin dependent kinases (CDKs).
